# Valorphins alter physicochemical characteristics of phosphatidylcholine membranes: Datasets on lipid packing, bending rigidity, specific electrical capacitance, dipole potential, vesicle size

**DOI:** 10.1016/j.dib.2022.108716

**Published:** 2022-11-02

**Authors:** Victoria Vitkova, Galya Staneva, Rusina Hazarosova, Stela I. Georgieva, Iva Valkova, Krassimira Antonova, Petаr Todorov

**Affiliations:** a Institute of Solid State Physics, Bulgarian Academy of Sciences, 72 Tzarigradsko Chaussee Blvd., 1784 Sofia, Bulgaria; b Institute of Biophysics and Biomedical Engineering, Bulgarian Academy of Sciences, Acad. G. Bonchev Str., bl. 21, Sofia 1113, Bulgaria; c Department of Analytical Chemistry, University of Chemical Technology and Metallurgy, 8 Kliment Ohridski Blvd., 1756 Sofia, Bulgaria; d Department of Organic Chemistry, University of Chemical Technology and Metallurgy, 8 Kliment Ohridski Blvd., 1756 Sofia, Bulgaria; e Department of Chemistry, Faculty of Pharmacy, Medical University, 2 Dunav Str. 1000 Sofia, Bulgaria

**Keywords:** Lipid vesicles, Fluctuation analysis, Isothermal titration calorimetry, Voltammetry, Laurdan, Di-8-ANEPPS, Aib, 2-aminoisobutyric acid, АTSF, analysis of thermal shape fluctuations, CV, cyclic voltammetry, Dab, 2,4-diaminobutanoic acid, Dap, 2,3-diaminopropanoic acid, Di-8-ANEPPS, 4-(2-[6-(Dioctylamino)-2-naphthalenyl]ethenyl)-1-(3-sulfopropyl)pyridinium inner salt, DPV, differential pulse voltammetry, Gln, glutamine (Q), GP, generalized polarization, GUV, giant unilamellar vesicle, Ile, isoleucine (I), ITC, isothermal titration calorimetry, ITO, indium tin oxide, LUV, large unilamellar vesicle, PC, phosphatidylcholine, PDMS, polydimethylsiloxane, POPC, 1-palmitoyl-2-oleoyl-*sn*-glycero-3-phosphocholine, Pro, proline (P), Thr, threonine (T), Trp, tryptophan (W), Tyr, tyrosine (Y), Val, valine (V), VV-hemorphin-5, Val-Val-Tyr-Pro-Trp-Thr-Gln-NH_2_

## Abstract

Endogenous hemorphins are being intensively investigated as therapeutic agents in neuropharmacology, and also as biomarkers in mood regulation, inflammation and oncology. The datasets collected herein report physicochemical parameters of 1-palmitoyl-2-oleoyl-sn-glycero-3-phosphocholine membranes in the presence of VV-hemorphin-5 (Val-Val-Tyr-Pro-Trp-Thr-Gln) and analogues, modified at position 1 and 7 by the natural amino acid isoleucine or the non-proteinogenic 2-aminoisobutyric, 2,3-diaminopropanoic or 2,4-diaminobutanoic amino acids. These peptides have been previously screened for nociceptive activity and were chosen accordingly. The present article contains fluorescence spectroscopy data of Laurdan- and di-8-ANEPPS- labelled large unilamellar vesicles (LUV) providing the degree of hydration and dipole potential of lipid bilayers in the presence of VV-hemorphin-5 analogues. Lipid packing is accessible from Laurdan intensity profiles and generalized polarization datasets reported herein. The data presented on fluorescence intensity ratios of di-8-ANEPPS dye provide dipole potential values of phosphatidylcholine-valorphin membranes. Vesicle size and electrophoretic mobility datasets included refer to the effect of valorphins on the size distribution and ζ-potential of POPC LUVs. Investigation of physicochemical properties of peptides such as diffusion coefficients and heterogeneous rate constant relates to elucidation of transport mechanisms in living cells. Voltammetric data of valorphins are presented together with square-wave voltammograms of investigated peptides for calculation of their heterogeneous electron transfer rate constants. Datasets from the thermal shape fluctuation analysis of quasispherical ‘giant’ unilamellar vesicles (GUV) are provided to quantify the influence of hemorphin incorporation on the membrane bending elasticity. Isothermal titration calorimetric data on the thermodynamics of peptide-lipid interactions and the binding affinity of valorphin analogues to phosphatidylcholine membranes are reported. Data of frequency-dependent deformation of GUVs in alternating electric field are included together with the values of the specific electrical capacitance of POPC-valorphin membranes. The datasets reported in this article can underlie the formulation and implementation of peptide-based strategies in pharmacology and biomedicine.

**Specifications Table**

**Table:** 

Subject	Materials Science
Specific subject area	Soft matter characterization; biomimetic self-assembling molecules; synthetic opioid peptides; liposomal formulations; lipid membrane deformability
Type of data	Tables and Figures
How the data were acquired	Data on VV-hemorphin-5 interactions with lipid membranes refer to bilayer lipid models produced by electroformation of giant unilamellar quasispherical lipid vesicles and extrusion of large unilamellar lipid vesicles (LiposoFast, Avestin, Ottawa, Canada). The peptides investigated herein were obtained by solid phase synthesis [1]. Bending elasticity data were acquired by means of phase-contract light microscopy (Axiovert 100, Zeiss, Germany) and analysis of thermal shape fluctuations (ATSF) of giant unilamellar quasispherical lipid vesicles [2]. Specific electrical capacitance data of POPC-valorphin membranes were obtained from frequency-dependent electrodeformation (ED) data
	of GUVs in alternating electric field [3]. Square-wave cyclic voltammetry (Metrohm 797, Switzerland; Pt and Ag/AgCl electrodes) was applied to evaluate the heterogeneous electron transfer rate constants of peptides [4]. Isothermal titration calorimetry in multiple injection mode (NanoITC calorimeter, TA Instruments, Lindon, UT, USA) provided the heat of LUV dilution in hemorphin solutions, and the peptide-lipid binding isotherms. Dynamic light scattering and laser Doppler electrophoresis of large unilamellar vesicles were acquired by Zetasizer Advance Series Instrument (Malvern Analytical, United Kingdom) with a 4 mW 632.8 nm sample illumination and detection at 173°. Fluorescence spectroscopy data of dye-labelled membranes (FP-8300, Jasco, MD, USA) refer to Laurdan spectra recorded from 390 to 600 nm upon excitation at 355 nm, and to di-8-ANEPPS fluorescence intensity ratio at 670 nm upon excitation at 420 nm and 520 nm [5].
Data format	Raw Data Analyzed Data
Description of data collection	The values of the bending modulus reported in the present article were calculated from ATSF data on populations of 6 to 13 GUVs accepted according to algorithms applied for elimination of systematic artifacts including correlated contours, volume and surface changes, non-quasisphericity, blurred contours, and membrane defects [2]. Fluorescence spectroscopy data were collected from 20 measurements of two different LUV preparations for each peptide investigated. Membrane capacitance values were reported based on the electrodeformation data of 9 to 30 GUVs. Prior to evaluation of reaction enthalpies and binding isotherms ITC data were corrected by the average area of dilution peaks [6]. Electrochemical data are reported as the mean value of three independent measurements. Data on average vesicle sizes were calculated from multi-angle dynamic light scattering signal acquired from six independent measurements. The same number of runs was performed to obtain the electrophoretic mobility datasets.
Data source location	Institute of Solid State Physics, Bulgarian Academy of Sciences, 72, Tzarigradsko Chaussee, Blvd, 1784 Sofia, Bulgaria 42°39′09.5″N 23°23′18.6″E
Data accessibility	Available with this article and also at: https://data.mendeley.com/datasets/gs6wxvcvs6/2
Related research article	V. Vitkova, G. Staneva, R. Hazarosova, St. Georgieva, I. Valkova, K. Antonova, P. Todorov, Interaction of new VV-hemorphin-5 analogues with cell membrane models, Coll. Surf. B, 220 (2022) 112896 https://doi.org/10.1016/j.colsurfb.2022.112896

## Value of the Data

• The data reported herein provide detailed knowledge on the effects of the endogenous opioid peptide valorphin and new VV-hemorphin-5 analogues on molecular organization and important physicochemical parameters of the lipid bilayer, which are relevant to membrane-mediated mechanisms of valorphin interaction with cells and subcellular structures.
• Biophysical and pharmacological research community can benefit from these datasets, which outline a comprehensive picture of structural, thermodynamic and mechanical properties of lipid membranes containing valorphins with nociceptive activity.
• The datasets in this article were collected from *in-vitro* studies on the interaction of new morphinomimetic peptides with model lipid systems. They can support the development of new peptide drug candidates with target specificity and pharmacokinetics tailored by amino acid or backbone modifications via incorporation of non-natural amino acids.
• The data on new valorphin analogues with nociceptive activity may be helpful in the development of a broad range of liposome-based applications including the design of novel drug carriers.

## 1 Objective

This data contribution is related to an original research article studying the interaction of new VV-hemorphin-5 (Val-Val-Tyr-Pro-Trp-Thr-Gln) analogues with cell membrane models. The accompanying Data-in-Brief material provides comprehensive information on the conducted experiments by adding complementary details about the applied methods, experimental protocols and data acquisition. The description of the experimental procedures is presented together with the raw and analyzed data supporting the discussion in the related research article. Furthermore, data available here are completed by data collections from fluorescence spectroscopy, isothermal titration calorimetry, voltammetry, and vesicle electrodeformation provided in a repository. The additional material presented herein is a necessary supplement, which makes accessible the grounds of conclusions regarding the effect of VV-hemorphin-5 and new valorphin analogues with nociceptive properties on lipid membranes’ structural organization, mechanical and electrical properties.

## 2 Data Description

Five datasets are provided in repository containing raw and analyzed data from Laurdan and di-8-ANEPPS fluorescence spectroscopy and electrokinetic measurements of POPC-valorphin LUV suspensions, POPC-valorphin GUV electrodeformation data as well as voltammetric data of valorphins with nociceptive activity (https://data.mendeley.com/datasets/gs6wxvcvs6/2). The reported parameters were acquired from measurements performed on membranes of 1-palmitoyl-2-oleoyl-sn-glycero-3-phosphocholine (POPC) containing VV-hemorphin-5 (Val-Val-Tyr-Pro-Trp-Thr-Gln-NH_2_) or its analogues, modified at position 1 and 7 by the natural amino acid isoleucine or the non-proteinogenic 2-aminoisobutyric, 2,3-diaminopropanoic or 2,4-diaminobutanoic amino acids. Peptide notation is reproduced from [1].

Dataset 1 contains analyzed data from Laurdan and di-8-ANEPPS fluorescence spectroscopy of POPC-valorphin LUV suspensions. The normalized spectra and general polarization data of Laurdan are included. The fluorescence intensity ratio of di-8-ANEPPS and the membrane dipole potential of POPC bilayers with all valorphins studied are reported.

Dataset 2 reports the analysed data from the frequency-dependent deformation of GUVs in alternating electric field for the specific electrical capacitance of POPC membranes in the presence of the studied peptides. The measured critical frequencies [7] of the AC field applied at which vesicles become quasispherical are summarized together with the vesicle radii and the conductivities of the aqueous solutions.

Dataset 3 provides analysed electrokinetic data, reporting the vesicles sizes and zeta potential values of POPC-valorphin LUV samples.

Dataset 4 comprises cyclic voltammetry data of valorphins in supporting electrolyte (0.1 mol L^−1^ phosphate buffer solution, pH 6.78 ± 0.01). Voltamperograms were recorded in square wave voltammetric (SWV) mode at 100 mV/s scan rate. Aliquots (50-100 µL) of the standard analyte solution were measured by subsequently adding them in the solution of the supporting electrolyte.

Dataset 5 reports isothermal titration calorimetry (ITC) injection heats Q of dilution of POPC LUVs in valorphin solutions. Control data were obtained from blank measurements in bidistilled water.

**Fig. 1:**
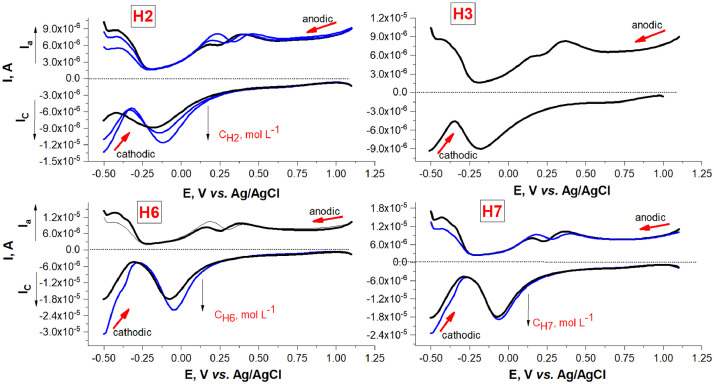
Square-wave voltammograms at Pt-working electrode of modified valorphins; pulse height $E_{sw}=$ 20 mV; scan increment ΔE=2 mV; frequency f= 50 Hz; scan rate ν= 100 mV/s.

Fig. 1 shows the voltammograms of the peptide compounds in anode and cathode mode at pH 6.78±0.01 (phosphatic buffer solution as supporting electrolyte). Well-formed current signals were obtained in both the anode and cathode potentials. The difference in the potentials of the cathodic ($E_{pc}$) and anodic ($E_{pa}$) peaks $\Delta E_{p}=\left|E_{pc}-E_{pa}\right|$ as well as the ratio $I_{pc}/I_{pa}$ between the cathode ($I_{pc}$) and anode ($I_{pa}$) current suggests quasi-reversibility of the electrode reaction for all compounds.

Table 1 summarizes the calculated coefficients of the function, representing the anodic current $I_{pa}=f\left(C,\;mol/L\right)$ calculated using the proportional increase of the concentration in the cathode mode. The heterogeneous electron transfer rate constants ($k_{sh}^{0}$) for all peptide derivatives are evaluated using the function expressing the cathodic current $I_{pc}=f\;\left(C,\;mol\;L^{-1}\right)$ and Reinmuth expression: $I_{pc}=nFACk_{sh}^{0}$ [4], where A is electrode area, n stands for the number of electrons transferred and F denotes Faraday's constant. The values of $k_{sh}^{0}$ for all compounds are summarized in Table 1. $k_{sh}^{0}$ values of the order of 10^−6^ cm/s characterize reversible or quasi-reversible reduction of peptides [8]. The diffusion coefficient D was calculated from Randles-Secik equation [9] at number of electrons transferred n=2 (from DPV [8]). Lower diffusion coefficients of H2, H3, and H6 compounds are obtained compared to H1.

**Table 1:** Equation of regression of $I_{pc}$ vs. concentration (C, mol L^−1^); heterogeneous rate constant and diffusion coefficient of H1-H3, H6 and H7 peptide compounds at Pt electrode (with electrode area, A=0.30 cm^2^).

Compound	Regression equation of $I_{pc}$, $A=f\;(C,\;mol\;L^{-1})$	$k_{sh}^{0}$, 10^−6^ cm/s	D, 10^3^ cm^2^/s
H1	$I_{p}=$ 7.42 × 10^−9^+ 0.0089(±0.0009) × C, R^2^=0.988	1.37	1.12
H2	$I_{p}=$1.35 × 10^−5^+ 0.311(±0.013) × C, R^2^=0.995	0.56	0.112
H3	$I_{p}=$1.29 × 10^−5^+ 0.336(±0.019) × C, R^2^=0.996	0.61	0.121
H6	$I_{p}=$1.29 × 10^−5^+ 0.327(±0.008) × C, R^2^=0.997	0.60	0.180
H7	$I_{p}=$1.33 × 10^−9^+ 0.283(±0.001) × C, R^2^=0.987	0.51	0.102

**Fig. 2:**
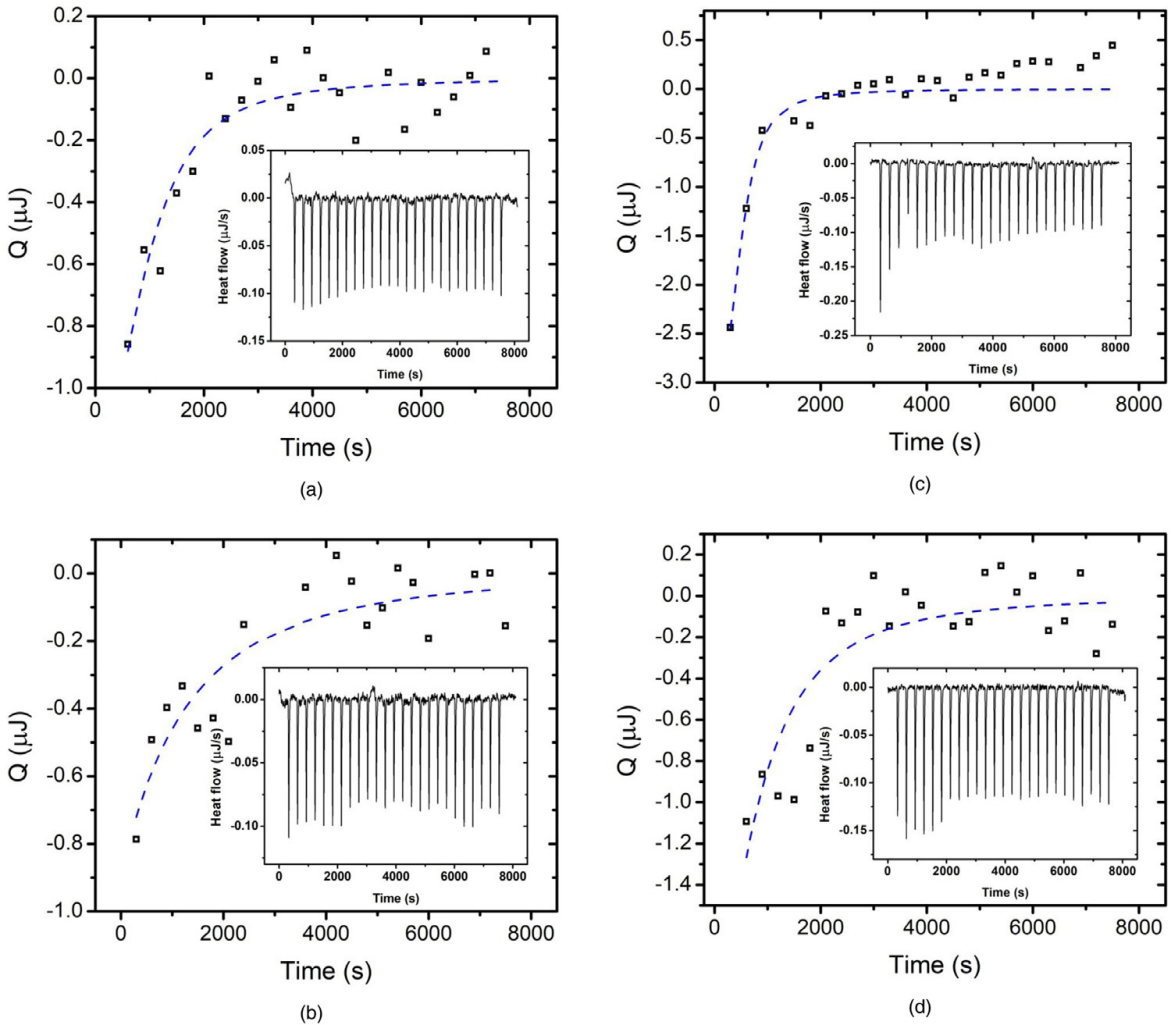
ITC results for the heat flows (inset graphics) and heats of reaction Q: (a) H1 (3.5 µmol L^−1^); (b) H2 (3.3 µmol L^−1^); (c) H3 (3.3 µmol L^−1^); (d) H6 (3.3 µmol L^−1^) VV-hemorphin-5 derivatives; 1 mmol L^−1^ POPC.

Fig. 2 represents thermodynamic parameters of valorphin-membrane interactions assessed on model systems of POPC LUV suspensions and diluted valorphin aqueous solutions with micromolar peptide concentrations. The heat of LUV dilution in the hemorphin solution was measured by ITC multiple injection mode. ITC measurements [10] were performed by NanoITC calorimeter (TA Instruments, Lindon, UT, USA) with volumes of the sample cell and the syringe 190 µL and 50 µL, respectively. LUV suspensions were prepared from POPC with concentration of 1 mmol L^−1^ in bidistilled water. Aqueous solutions of valorphins contained final peptide concentration of 0.0033 mmol L^−1^. All samples were degassed prior to experiment. Upon measurement the syringe contained LUV suspension, while the peptide solution was placed in the sample cell. Titration was performed by 2 µL aliquots in 25 steps at 300 s intervals while stirring at 22 °C. The heat of LUV dilution in bidistilled water was determined in a blank experiment at the same conditions. NanoAnalyze software (TA Instruments, Lindon, UT, USA) was used to process the data and calculate the thermodynamic parameters of interaction.

Table 2 presents ATSF data obtained for the radius $R_{ves}$, the bending constant $k_{c}$, and the reduced membrane tension $\bar{\sigma }=\sigma R^{2}/k_{c}$ of all recorded and analyzed GUVs [2,^11^,12]. The values of the membrane bending modulus were obtained over an ensemble of not less than six GUVs collected from at least three different electroformation batches. All $k_{c}$ values reported were calculated following the fitting procedure described in [2,12] from ATSF of GUVs complying with the eligibility criteria previously established, including the absence of membrane defects and heterogeneities; constant vesicle volume during measurements; uniformity of the mean radius of the vesicular contour over all angular directions; non-correlated images taken for analysis for each studied vesicle. For the six GUVs populations, including a POPC control set and five POPC-valorphin compositions given in Table 1, the best data fit was calculated by $\chi^{2}$ minimization.

**Table 2:** ATSF data for the radius $R_{ves}$, membrane bending modulus $k_{c}$, and tension $\bar{\sigma }$ of POPC-valorphin membranes; GF – goodness of fit ($\chi^{2}$-test); the total peptide-to-lipid molar ratio P/L in the sample is reported in mol/mol; next to the batch description the bending modulus calculated for each POPC-valorphin membrane composition as the weighted average with standard deviation is given.

$R_{ves}$, µm	$k_{c}$, $10^{-19}$J	$\bar{\sigma }$	GF
*Control, POPC, H_2_O, pH 5.6,*$k_{c}=\left(1.46\pm 0.05\right)10^{-19}$*J*			
7.43	1.84±0.48	16.50±0.90	0.43
11.54	1.34±0.18	-0.30±0.40	0.28
7.94	1.54±0.18	1.60±0.90	0.56
5.44	1.36±0.15	-3.40±0.60	0.61
7.88	1.51±0.18	1.90±1.80	0.39
6.68	1.97±0.79	24.00±16.00	0.62
6.57	2.16±1.25	35.00±27.00	0.26
7.66	2.43±1.14	48.00±29.00	0.45
*10^−2^ mol/mol H1/POPC, H_2_O, pH 5.6;*$k_{c}=\left(1.67\pm 0.08\right)10^{-19}$*J*			
9.46	1.56±0.31	69.00±25.00	0.64
11.31	1.58±0.68	133.00±74.00	0.63
7.61	1.74±0.27	8.00±5.00	0.46
9.83	1.45±0.41	-1.50±0.80	0.29
11.44	1.77±0.48	31.00±16.00	0.49
6.48	1.31±0.43	3.50±2.40	0.30
7.64	2.19±0.89	47.00±26.00	0.38
6.49	1.67±0.50	22.00±10.00	0.45
7.05	2.11±1.19	26.00±22.00	0.55
15.28	2.39±0.81	73.00±32.00	0.67
16.34	3.44±2.34	137.00±105.00	0.35
7.42	2.24±1.45	51.00±42.00	0.41
12.52	3.58±2.56	80.00±67.00	0.42
*10^−2^ mol/mol H2/POPC, H_2_O, pH 5.6;*$k_{c}=\left(1.46\pm 0.09\right)10^{-19}$*J*			
10.69	2.20±0.64	113.00±45.00	0.50
9.24	2.12±0.41	38.00±13.00	0.25
5.49	1.60±0.29	-5.00±0.40	0.29
7.72	1.86±0.64	41.00±19.00	0.56
5.33	1.52±0.52	-2.00±1.00	0.18
15.71	3.87±2.30	77.00±55.00	0.48
16.30	1.41±0.22	-0.30±1.00	0.48
12.71	1.42±0.15	3.50±1.60	0.29
9.64	1.28±0.17	-2.80±0.60	0.18
9.22	1.66±0.79	19.00±15.00	0.52
*10^−2^ mol/mol H3/POPC, H_2_O, pH 5.6;*$k_{c}=\left(1.17\pm 0.11\right)10^{-19}$*J*			
6.23	0.97±0.20	-4.80±0.20	0.65
10.94	2.76±0.91	52.00±44.00	0.43
17.16	2.78±0.94	27.00±15.00	0.31
10.34	1.32±0.56	121.00±89.00	0.52
8.28	1.44±0.42	46.00±27.00	0.27
6.88	1.15±0.19	16.10±9.80	0.39
*10^−2^ mol/mol H6/POPC, H_2_O, pH 5.6;*$k_{c}=\left(1.44\pm 0.09\right)10^{-19}$*J*			
8.76	1.37±0.13	-1.80±0.80	0.19
9.78	1.38±0.72	75.00±56.00	0.54
6.66	1.50±0.39	10.00±6.00	0.22
6.59	4.42±2.31	10.00±9.00	0.34
8.39	2.57±0.97	40.00±20.00	0.71
11.99	2.88±0.88	46.00±19.00	0.47
*10^−2^ mol/mol H7/POPC, H_2_O, pH 5.6;*$k_{c}=\left(1.39\pm 0.12\right)10^{-19}$*J*			
10.34	2.15±0.59	43.00±19.00	0.41
6.11	4.39±3.05	19.00±20.00	0.42
11.54	1.95±1.42	116.00±97.00	0.23
10.48	2.40±0.72	46.00±21.00	0.46
5.24	1.26±0.43	6.10±5.70	0.30
5.70	1.26±0.23	-4.50±0.40	0.27
5.76	1.15±0.40	6.70±5.70	0.62

## 3 Experimental Design, Materials and Methods

Giant unilamellar vesicles (GUVs) were obtained in electroformation cells, consisted of two indium tin oxide (ITO)-coated glass plates and a polydimethylsiloxane (PDMS, Dow Corning, Germany) spacer [13]. A small quantity (∼50 μL) of POPC-valorphin mixture with total concentration of 1 g L^−1^ in chloroform-methanol (Sigma Aldrich, Germany) solvent (9:1 volume parts) was uniformly applied on the ITO-coated side of the electrodes. All traces of organic solvents were evaporated under vacuum. Afterwards, the electroformation chamber (∼4 mL) was entirely filled up with bidistilled water or with 10 mmol L^−1^ NaCl aqueous solution, which had been previously degassed under vacuum. AC electric field with frequency 10 or 500 Hz, depending on the conductivity of the aqueous medium, and the peak-to-peak voltage, was successively increased to 4 V. A high yield of unilamellar quasispherical vesicles appropriate for analysis was obtained in several hours. The conductivities of the aqueous media were measured by Cyberscan PC510 (Eutech, Singapore). Prior to GUV electrodeformation experiments 0.1 mM NaCl was added to vesicle suspensions according to the method requirements towards the conductivity of the external aqueous solution [7].

Laurdan and Di-8-ANEPPS fluorescence spectroscopy measurements were carried out on suspensions of large unilamellar vesicles (LUV), obtained by a LiposoFast small-volume extruder equipped with polycarbonate filters (Avestin, Ottawa, Canada) [5]. Laurdan-labelled samples contained 1:200 probe:lipid molar ratio. Di-8-ANEPPS was added to the lipid-peptide organic solution at 1:250 probe-to-lipid molar ratio with lipid concentration of 1 mM. The lipid film was formed on the flask bottom by removing the organic solvent under a stream of oxygen-free dry nitrogen followed by desiccation under vacuum overnight. Subsequently, filtered (0.2 µm) bidistilled water was added to achieve the lipid concentration of 1 mM. The samples were vortexed for 1 min, and then left in a sonication bath for 5 min. The multilamellar vesicles obtained at this stage were then extruded 11 times through 800 nm filters, followed by 21 extrusions through 100 nm filters.

### 3.1 Fluorescence spectroscopy of Laurdan- and di-8-ANEPPS-labelled LUVs

Lipid packing in the bilayer was estimated by calculating Laurdan generalized polarization parameter $GP=\left(I_{440}-I_{490}\right)/\left(I_{440}+I_{490}\right)$, with $I_{440}$ and $I_{490}$, which denote the intensities of the emission light at 440 nm and 490 nm, respectively. The excitation wavelength for fluorescent probe was 355 nm and emission was recorded from 390 to 600 nm. For each LUV suspension studied the final lipid concentration in the quartz cuvette was 200 µmol L^−1^. The measurements were performed the same day at 22±1°C. Each sample was measured 10 times and averaged by three different LUV preparations after background subtraction.

The dipole potential of dye di-8-ANEPPS-labelled POPC-valorphin membranes was measured by fluorescence spectroscopy of di-8-ANEPPS excited at 420 nm and 520 nm and detected at 670 nm. Subsequently, the fluorescence intensity ratio $R_{\text{ex}}=I_{670\;(\text{exc}.420)}/I_{670\;(\text{exc}.520)}\;$and the dipole potential $\Psi_{d}=\left(R_{\text{ex}}+0.3\right)/0.0043$, [14], [15], [16] were calculated. All measurements were performed at a total lipid concentration of 200 µmol L^−1^ in a thermostated ($\pm 0.1^{\circ }C$) cuvette holder of FP-8300 spectrofluorometer (Jasco, MD, USA) with excitation and emission slits adjusted to 5 nm.

### 3.2 Electrochemical measurements

Stock standard solutions of VV-hemorphin-5 derivatives were prepared from dry pure substances in methanol:water (v/v) = 1:1 and with concentration as follows: 1.00 mmol L^−1^ H1, 0.861 mmol L^−1^ H2, 0.959 mmol L^−1^ H3; 0.889 mmol L^−1^ H6; 1.09 mmol L^−1^ V7, respectively. Aqueous phosphate buffer solutions at pH 6.86 (0.1 mol L^−1^) was prepared from potassium dihydrogen phosphate and potentiometrically controlled with digital pH-meter Jenway (Cole-Parmer, UK) by adding aqueous solution of NaOH (0.1 mol L^−1^). Potassium dihydrogen phosphate KH_2_PO_4_ and sodium hydroxide NaOH were supplied by Sigma-Aldrich (Germany). The presented results were reported as the mean value of three independent measurements. Prior to measurement the working electrode was rinsed thoroughly in deionised water and electrochemically cleaned by potential cycling in 0.5 mol L^−1^ sulphuric acid and stored in 98% sulphuric acid. The solution in the electrochemical cell was degassed under high purity nitrogen for 10 min.

The analytical signal of the supporting electrolyte (0.1 M phosphate buffer solution, pH 6.78 ± 0.01) in the electrochemical cell was recorded on Metrohm 797 (Switzerland) VA trace analyzer with a Pt-disk working electrode (3 mm^2^), Ag/AgCl (3 mol/L KCl) reference electrode and a Pt-wire counter electrode. Voltammetric experiments were conducted in a high purity nitrogen atmosphere at 25±1°C. The voltamperogram of the blank sample was reordered in square-wave voltammetric mode with 50 Hz frequency and 2 mV potential increments at 100 mV/s scan rate. Under the same conditions the required aliquots of standard analyte solution (50-100 µL) were measured by subsequently adding them in the solution of the supporting electrolyte.

### 3.3 Isothermal titration calorimetry

Data on thermodynamic parameters of valorphin-membrane interactions were acquired by a NanoITC calorimeter (TA Instruments, Lindon, UT, USA) with a reaction cell of 190 µL and 50 µL syringe at 22 °C. A final valorphin concentration of 3.3 µmol L^−1^ in bidistilled water was prepared. All samples were degassed prior to experiment. The peptide solution was placed in the sample cell, while the syringe contained LUV suspension in water with total lipid concentration of 1 mmol L^−1^. LUV suspension as aliquots of 2 µL was injected in 25 steps with 300 s intervals at stirring speed of 250 rpm at 25 °C. The heat of LUV dilution in bidistilled water was determined also and served as a control. Changes in the heat rate were registered, processed by the ITCRun software to determine the injection heats by an integration procedure. NanoAnalyze software (TA Instruments, Lindon, UT, USA) was used to process the data and quantify the thermodynamic parameters of interaction.

### 3.4 Bending elasticity data of POPC GUVs in the presence of valorphins

Measurements of the bending elasticity of POPC membranes were performed by thermal shape fluctuation analysis (TSFA) according to [2]. All measurements were carried out at 22°C. Monitoring and recording were performed by an inverted microscope Axiovert 100 (Zeiss, Germany) in phase contrast regime, using an oil-immersed objective Zeiss N-Achroplan (Ph3, 100x, NA 1.25). Observation chamber consisted of two parallel glass slides, separated by a 0.5 mm-thick (CoverWell®) spacer (Sigma-Aldrich Inc., USA). The internal volume contained 400 µL of a GUV suspension freshly prepared. Flaccid, nearly spherical vesicles with diameters of the order of 10 μm and larger were recorded and subsequently subjected to TSFA. Several hundred images were acquired once per second. After image treatment and quality assessment [2] the membrane bending constant and tension of every studied vesicle were determined via Legendre analysis of the autocorrelation function of the vesicle contour [12,17]. For the calculation of the bending modulus reported here as the weighted average with standard deviation, only vesicles satisfying all selection criteria for quality [2] were considered.

### 3.5 GUV electrodeformation measurements

Frequency-dependent deformation data of GUVs in alternating electric field were collected upon decreasing the AC field frequency. Morphological changes of a vesicle with radius a placed in aqueous medium with conductivity $\lambda_{out}>\lambda_{in}$ ($\lambda_{in}$ stands for the conductivity of the aqueous solution enclosed by the vesicle membrane) were recorded and analysed. The frequency, $f_{cr}=\frac{\lambda_{in}}{2\pi a{\bar{C}}_{m}}{\left[\left(1-\Lambda )(\Lambda +3\right)\right]}^{-1/2}$, at which the vesicle assumed quasispherical shape, was measured [7,18]. Knowing $\Lambda =\lambda_{in}/\lambda_{out}$ and a and fitting with the above equation the experimental data $f_{cr}\left(a^{-1}\right)$ for a batch of 9 to 30 GUVs from at least two different preparations we calculated the resultant capacitance ${\bar{C}}_{m}={\left(1/C_{m}+1/C_{D,in}+1/C_{D,ex}\right)}^{-1}$, including the lipid bilayer, $C_{m}$, and the capacitances $C_{D,in}$ and $C_{D,ex}$ of the diffuse charge regions, formed in the aqueous surroundings at the two sides of the bilayer.

Electrodeformation measurements were carried out at 22°C in a cell consisted of two parallel glass slides separated by a 0.5 mm-thick inert spacer (Sigma-Aldrich Inc., St Louis, MO, USA). The AC electric field was obtained from an arbitrary waveform generator (33120A, HP/Agilent, Santa Clara, CA, USA), connected to a pair of rectangular parallel ITO-electrodes deposited on the lower glass slide at 1 mm apart. In all measurement the frequency of the imposed uniform field with strength ≤ 7 kV/m was in the range of 10–200 kHz. Vesicles were observed and analysed the day of preparation by means of a phase-contrast microscope (В-510PH, Optika, Italy) operating with a dry objective (× 40, NA 0.65) and Axiocam ERc 5s camera 5 MP (Zeiss, Germany) with resolution of 0.1 μm/pixel. The bulk conductivity ratio Λ=0.96 in conducted experiments corresponded to more conductive suspending medium. Data analysis was performed following Salipante et al. [7,19].

### 3.6 Vesicle size and electrophoretic mobility

LUV size distribution was assessed by dynamic light scattering measurements on a Zetasizer Advance Series Instrument (Malvern Analytical, United Kingdom) equiped with a 4 mW HeNe laser (632.8 nm). The detection was performed at an angle of 173°. Laser Doppler electrophoresis of LUVs was applied to measure the speed of liposomes in aqueous phase upon application of electric field. Zeta potential $\zeta =\frac{\eta u}{\varepsilon_{r}\varepsilon_{0}}$ [20] was calculated from the electrophoretic mobility, u, measured, and the known relative dielectric permittivity of the aqueous phase $\varepsilon_{r}$, the vacuum permittivity $\varepsilon_{0}$, and the water viscosity η.

## Ethics Statements

Not applicable. These data does not include data from experiments with any human subjects, animal experiment, or social media platforms.

## CRediT Author Statement

**Victoria Vitkova:** Conceptualization, Methodology, Investigation, Data curation, Writing – original draft preparation; **Galya Staneva:** Data curation, Visualization, Writing – review & editing; **Rusina Hazarosova:** Visualization, Investigation; **Stela Georgieva:** Visualization, Investigation; **Iva Valkova:** Visualization, Investigation; **Krassimira Antonova:** Investigation; **Petar Todorov:** Conceptualization, Resources.

## Declaration of Competing Interest

The authors declare that they have no known competing financial interests or personal relationships that could have appeared to influence the work reported in this paper.

## Data Availability

Data from fluorescence spectroscopy, isothermal titration calorimetry, voltammetry, and vesicle electrodeformation for characterisation of lipid membranes in the presence of VV-hemorphin-5 analogues (Original data) (Mendeley Data).
